# An Ensemble Learning Method for Robot Electronic Nose with Active Perception

**DOI:** 10.3390/s21113941

**Published:** 2021-06-07

**Authors:** Shengming Li, Lin Feng, Yunfei Ge, Li Zhu, Liang Zhao

**Affiliations:** 1 School of Computer Science and Technology, Dalian University of Technology, Dalian 116024, China; lishengming@dlut.edu.cn (S.L.); fenglin@dlut.edu.cn (L.F.); katsuunhi@gmail.com (Y.G.); 2 School of Innovation and Entrepreneurship, Dalian University of Technology, Dalian 116024, China; 3 Key Laboratory of Intelligent Control and Optimization for Industrial Equipment of Ministry of Education, Dalian University of Technology, Dalian 116024, China; zliang@dlut.edu.cn; 4 School of Control Science and Engineering, Dalian University of Technology, Dalian 116024, China

**Keywords:** electronic nose, ensemble learning, active perception

## Abstract

The electronic nose is the olfactory organ of the robot, which is composed of a large number of sensors to perceive the smell of objects through free diffusion. Traditionally, it is difficult to realize the active perception function, and it is difficult to meet the requirements of small size, low cost, and quick response that robots require. In order to address these issues, a novel electronic nose with active perception was designed and an ensemble learning method was proposed to distinguish the smell of different objects. An array of three MQ303 semiconductor gas sensors and an electrochemical sensor DART-2-Fe5 were used to construct the novel electronic nose, and the proposed ensemble learning method with four algorithms realized the active odor perception function. The experiment results verified that the accuracy of the active odor perception can reach more than 90%, even though it used 30% training data. The novel electronic nose with active perception based on the ensemble learning method can improve the efficiency and accuracy of odor data collection and olfactory perception.

## 1. Introduction

With the development of the Internet of Things, it is increasingly more necessary to realize olfactory perception in mobile robots [1]. Odor is an external characteristic of substances and can accurately represent their essence [2]. It is more reliable to use odor for target recognition. Therefore, research on the electronic nose and machine olfaction is also increasing year by year [3].

Thus far, there have been many studies and attempts on machine olfaction to solve the drift phenomenon of electronic noses [4] and to achieve classification learning of machine olfaction. Zhu [5] presented a system for the evaluation of Oolong tea odor with sensory analysis and an E-nose. Hu [6] evaluated the sensory quality of Prinsepia utilis tea, using E-nose/E-tongue techniques. They all used traditional olfactory algorithms to process, and they ultimately achieved some simple applications of olfaction, but this is not enough for complex scenes of robots. Zhang [7] used domain adaptation extreme learning machines to solve the problem of data drift in electronic nose acquisition. It eliminated the drift effect of the electronic nose and provided a good idea for data acquisition of the electronic nose. The main disadvantage of this research is that it still used the natural diffusion method to collect data, which made the data acquisition time too long. A piece of data took about 15–20 s. In recent studies, Falasconi et al. [8] finally achieved a 94% accuracy based on cluster analysis. Brahimi-Belhouari et al. [9] applied the Gaussian mixture model (GMM), probabilistic principal component analysis mixture model, and the generative graph classifier to obtain the results for the target gas of methane, carbon monoxide, hydrogen, and their mixtures, and high accuracies of 94.5% and 96% could be achieved within 15 and 30 s, respectively. Although high accuracy was acquired, the time cost was still too long, which is not suitable for robots.

By analyzing previous research work, it is found that the time taken for data collection in most studies is more than 15 s, and the time of the data collection method based on free diffusion is longer. In addition, in-depth learning requires a large number of datasets, which also increases the data preparation time. Although the requirement of data quantity for a single machine learning algorithm is reduced, there is still room for improvement of accuracy. If these problems can be solved, the implementation of olfactory perception will be more convenient and faster, and the results will be more reliable.

In this paper, a novel electronic nose with active perception was designed, which consisted of three semiconductor sensors and an electrochemical sensor array. Meanwhile, the STM32F334 microprocessor was used to control the air duct to capture odor. The STM32F334 is an ARM Cortex-M4 32-bit CPU with an FPU (72 MHz Max), manufactured by STMicroelectronics (Grenoble, France). It has two ADC 0.20 s (up to 21 channels) with a resolution of 12 bits, a 0 to 3.6 V conversion range, and a 7-channel DMA controller, which are suitable for electronic nose systems. Besides, an ensemble learning method was proposed to distinguish the smell of the different objects. This proposed ensemble learning method was not only used for the numerous training data, but also suitable for the small-size sampling data. The main contributions are as follows:

(1) The designed electronic nose can actively perceive the different smells.
(2) The designed electronic nose with active perception reduces the time taken for data acquisition.
(3) The proposed ensemble learning method can not only realize active perception, but also solve the problem of the small training data.

The content of this paper is organized as follows. In Section 2, the hardware design experiment of the electric nose is described, and the recognition method of odors based on the ensemble learning method is presented in Section 3. The designed electronic nose with active perception is validated by the experiment and the results are discussed in Section 4. Finally, the conclusions are drawn in Section 5.

## 2. Hardware Design of Electric Nose

The gas sensor is the core component of odor information acquisition. For example, in a civil electronic nose system, a contact combustion semiconductor gas sensor array such as a TGS2620 can be used to realize odor sensing. The semiconductor gas sensor is made by using the REDOX reaction of gas on the semiconductor surface, which leads to the change in the resistance value of the sensitive element. It has the advantages of fast response and low price, but poor selectivity. In recent years, electrochemical sensors have been widely used in the Internet of Things, environmental monitoring, chemical industry, steel and other industrial fields due to their good selectivity and high sensitivity [10]. Electrochemical gas sensors work by reacting with the gas to be measured and producing an electrical signal proportional to the concentration of the gas. However, it is expensive and requires proper circuitry to work well.

In order to improve the response speed and measurement accuracy, the electronic nose system adopted the combination of a semiconductor gas sensor and electrochemical gas sensor. Compared with the traditional electronic nose system with an abundant gas sensor array, the designed electronic nose system only used four gas sensors, which is composed of an electrochemical sensor DART-2-Fe5 and three MQ303 semiconductor gas sensors, to reduce the cost. DART-2-FE5 is an electrochemical gas sensor produced by the DART Company in the UK. It can be continuously monitored and has the advantages of high precision, low power consumption, and small volume. MQ303 is a tin dioxide semiconductor gas sensor for portable systems produced by WinSensor (Zhengzhou, China), a Chinese company. It has the advantages of high sensitivity and fast response.

**Figure 1:**
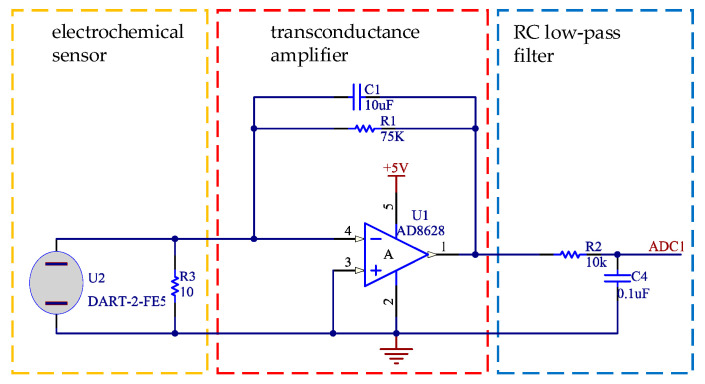
Circuit of electrochemical gas sensor.

The working circuit of the sensor DART-2-FE5 was designed as shown in Figure 1.

The electrochemical sensor uses the electrochemical oxidation process of the gas on the electrode. Accompanying the electrochemical reaction, the generated current is proportional to the gas concentration. As a result, the gas concentration can be determined by measuring the current. In Figure 1, R3 acts as the load of sensor current output. R1 is the resistance of the transconductance amplifier, whose resistance value determines the output voltage. The transconductance amplifier amplifies the weak current signal of the electrochemical sensor, so the noise and bandwidth of the amplifier are required to be high. In order to improve the accuracy of the circuit, an operational amplifier AD8628 with ultra-low offset, drift, and bias current characteristics was adopted. After the amplifier converts the current signal into the voltage signal, a low-pass filter composed of R2 and C4 is input to the analog-to-digital converter for measurement. The cut-off frequency (*f_c_*) of the *RC* low-pass filter is calculated as shown in Equation (1).

(1) $$f_{c}=\frac{1}{2\pi RC}=\frac{1}{2\pi \times 0.01\Omega \times 0.1\mathrm{uF}}=159.2\mathrm{Hz}$$

**Figure 2:**
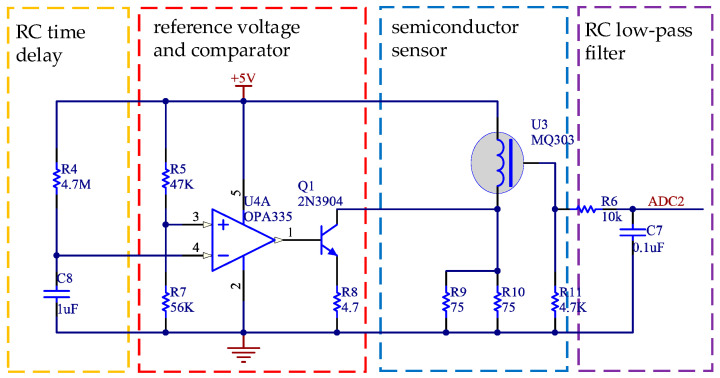
Circuit of semiconductor gas sensor.

The gas-sensitive part of semiconductor gas sensor is a microsphere with heating wire and a metal electrode embedded. The sensor is installed in the metal shell of the stainless-steel mesh. Semiconductor gas sensors usually need several minutes of preheating to enter a stable working state after electrification. We can also apply 3–10 s of 2.2 ± 0.2 V high voltage to the sensor before normal detection so the sensor can stabilize and enter the working state as soon as possible. The working circuit of MQ303 is designed in the system as shown in Figure 2.

The internal heating resistance of MQ303 is 4.5 Ω, and the heating current is 120 ± 20 mA. In order to make the sensor work as soon as possible, the *RC* time delay circuit and comparator are combined to provide a high voltage for the sensor when it is powered on. The reference voltage (Vref) at the whole end of the comparator is:

(2) $$Vref=\frac{R7}{R5+R7}\times Vcc=\frac{56K\Omega }{47K\Omega +56K\Omega }\times 5V=2.72V$$

In Figure 2, R4 and C8 constitute the *RC* charging circuit. The calculation formulas of charging time (T) and power supply voltage (Vcc), charging voltage (Vo), resistance (R), and capacitance (C) are as follows.

(3) $$T=-\ln\frac{Vcc-Vo}{Vcc}\times R\times C$$

The charging voltage of the *RC* charging circuit is the reference voltage (Vref), so the high voltage working time of the sensor is:

(4) $$T=-\ln\frac{5V-2.72V}{5V}\times 4.7M\Omega \times 1\mathrm{uF}=3.69S$$

The traditional electronic nose system uses the passive method of free gas diffusion to obtain gas information. The detection time is usually tens of minutes or even hours. In order to enable the robot to quickly collect the odor of the object to respond, the system uses the active adsorption method to collect gas information by designing the air duct to simulate the operation of nose breathing. A wind drum is driven by a DC motor blade to achieve air inflow and outflow. The DC motor drive control circuit is as follows.

**Figure 3:**
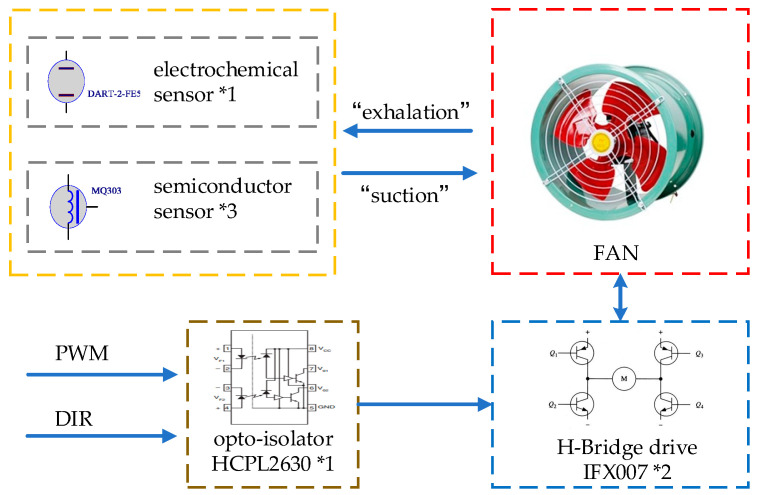
Driving circuit of windmill motor.

In Figure 3, the DC motor drive circuit consists of two IFX007 half-bridge chips, which can control the forward and reverse of the motor. The speed and direction of the motor are controlled. The isolation is realized by the photocoupler chip HCPL2630. The controller can adjust the speed and direction of the DC motor only by providing control signals PWM and DIR.

**Figure 4:**
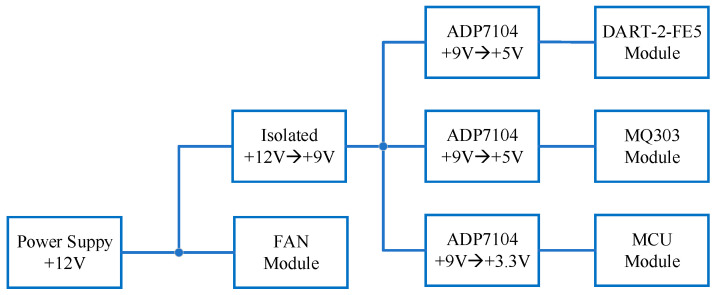
Power tree of olfactory acquisition circuit.

As an inductive component, the DC motor has a great influence on the system power supply when it works. In order to avoid the influence of the air sensor when the air duct works, besides the isolation of the control signal, the air duct power supply and the sensor power supply are separated from each other. The power supply of the sensor is supplied by isolated power, while the LDO chip ADP7104 with a low voltage drop and ultra-low noise is used to supply power for the working part of the sensor. The power tree structure of the system is shown in Figure 4.

The gas sensor circuit converts odor into a voltage signal, and the STM32F334 microprocessor performs ADC (analog-to-digital conversion). The acquisition process simulates human breathing action [11]. The acquisition process once is divided into the following steps:

(1) 500 ms DC motor reverses; at this time, the wind blows out from the wind drum to achieve ‘exhalation’;
(2) 2000 ms DC motor is forward; at this time, the wind is sucked into the air cylinder from outside to realize ‘suction’;
(3) 500 ms DC motor reverses; at this time, the wind blows out from the wind drum, to achieve ‘exhalation’.

Each channel collects 100 values per second and 300 values at a time. Each value takes the 12-bit effective value. The 300 collection points will take 3 s. STM32F334 communicates with Jetson TX2 through the serial port to transmit data and commands. Jetson TX2 is designed by NVIDIA for AI computing. This supercomputer module uses the NVIDIA Pascal™ GPU, up to 8 GB of memory, 59.7 GB/s of memory bandwidth, and has a rich standard hardware interface, so it is very suitable for electronic nose algorithm calculation and embedding. The program design of STM32F334 uses the embedded C language, MDK-ARM software platform, and Windows 10 64 bit. Jetson TX2 is installed on Ubuntu 18.04 LTS, with Python 3.0 and CUDA 8.0.

**Figure 5:**
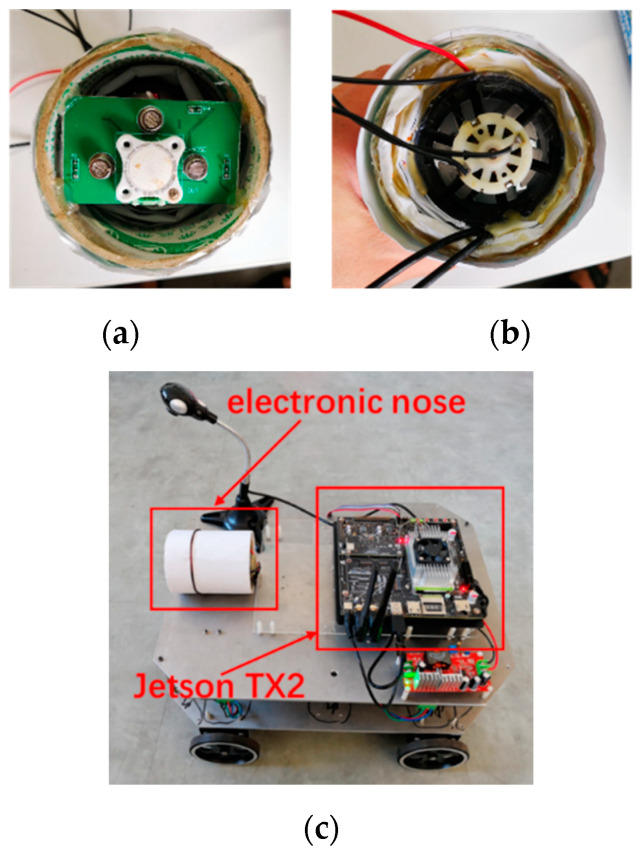
Front view of the active electronic nose (**a**), rear view (**b**), and installation on the robot (**c**).

The physical object of the electronic nose system is shown in Figure 5.

## 3. Ensemble Learning Method

Ensemble learning is a machine learning paradigm [12]. It is common to train multiple models to solve the same problem and combine them to achieve better results. The most important assumption is that when weak models are combined correctly, accurate and/or robust models are obtained. In the ensemble learning method, the basic models can be used as components to design more complex models. In most cases, the performance of these basic models themselves is not very good, either because they have high bias or because their variance is too large to be robust. The idea of the ensemble learning method is to create a strong learner by combining the basic models to achieve a better performance. In this paper, the decision tree, random forests, gradient boosting, Xgboost, SVM, and Gaussian process were used as the basic models, and the proposed ensemble learning method integrated these basic models to design complex models for better recognition performance.

The decision tree algorithm is a mature algorithm and has been applied to various scenarios [13]. The key of decision tree learning is to select the optimal partition attributes. With the partition proceeding, the samples contained in the branch nodes of the decision tree belong to one category gradually, that is, the ‘purity’ increases. In order to express the level of ‘purity’, decision tree mainly uses two measures: information gain and Gini index. The greater the information gain and the smaller the Gini index, the higher the purity. Here, we partitioned olfactory data based on information gain, executed the above algorithm, and input training data to train our weak classifier of decision tree.

Random forest [14] is an integration algorithm based on decision tree. It uses a subset of training data and a subset of variables to build multiple decision trees in a random way. The decision trees are independent of each other. When new inputs are available, each decision tree is allowed to make decisions. Finally, the final decision results are synthesized to be more accurate and stable.

The gradient boosting algorithm [15] consists of initialization, calculation of negative gradient, minimization of error, determination of step size, and output. The olfactory data are divided into M parts to train m base learners. Then, the negative gradients are calculated and fit with the base learner by minimizing the square error. The line search is used to determine the step length to minimize loss to obtain the output as:

(5) $$F_{m}\left(x\right)=F_{m-1}\left(x\right)+\rho_{m}h\left(x:a_{m}\right)$$

The function of the model is updated directly here, and the parameter additivity is extended to the function space. The trained *m* base learners keep updating $\rho_{m}$ and $a_{m}$ in the direction of gradient descent.

Xgboost is an efficient implementation of the gradient boosting algorithm [16]. Xgboost adds a regularization term to the objective function. When the base learning is CART, the regularization term is related to the number of leaf nodes *T* and the value of leaf nodes. Meanwhile, xgboost uses first and second derivatives to compute pseudo-residuals for learning generation. For Taylor expansion of the loss function, the following formulas are obtained, where *g* is the first derivative and *h* is the second derivative,

(6) $$L^{\left(t\right)}≃\sum_{i=1}^{n}\left[l\left(y_{i},\;{\hat{y}}^{\left(t-1\right)}\right)+g_{i}f_{t}\left(X_{i}\right)+\frac{1}{2}h_{t}f_{t}^{2}\left(X_{i}\right)\right]+\Omega \left(f_{t}\right)$$

$\mathrm{where}\;g_{i}=\partial_{{\hat{y}}^{\left(t-1\right)}}l\left(y_{i},{\hat{y}}^{\left(t-1\right)}\right)\;and\;h_{i}=\partial_{{\hat{y}}^{\left(t-1\right)}}^{2}l\left(y_{i},{\hat{y}}^{\left(t-1\right)}\right)$. The criterion to find dividing points of xgboost is maximization. λ and γ are related to the regularization term. The calculation formula is as follows,

(7) $${ℒ}_{split}=\frac{1}{2}\left[\frac{{\left(\sum_{i\in I_{L}}g_{i}\right)}^{2}}{\sum_{i\in I_{L}}h_{i}+\lambda }+\frac{{\left(\sum_{i\in I_{R}}g_{i}\right)}^{2}}{\sum_{i\in I_{R}}h_{i}+\lambda }-\frac{{\left(\sum_{i\in I}g_{i}\right)}^{2}}{\sum_{i\in I}h_{i}+\lambda }\right]-\gamma$$

For the olfactory training set, a hyperplane is found [17] to partition the training set so that the Euclidean distance between different partitions of olfactory data to the hyperplane is maximum. The representation of the distance is shown in Equation (8). What we need to do is maximize ${\left|\left|w\right|\right|}^{-1}$, which is to minimize ${\left|\left|w\right|\right|}^{2}$:

(8) $$\begin{array}{c} \underset{w,b}{\min}\frac{1}{2}{\left|\left|w\right|\right|}^{2}\\ s.t.\;y_{i}\left(w^{T}x_{i}+b\right)\geq 1,\;i=1,2,\ldots ,m \end{array}$$

We must input olfactory data for multi-round training and constantly modify the parameters to minimize ${\left|\left|w\right|\right|}^{2}$ so that the final SVM weak classifier is obtained.

The Gaussian process [18] is a kind of stochastic process in probability theory and mathematical statistics. It is a combination of a series of random variables obeying a normal distribution in an exponential set. In the general Gaussian distribution model, the probability is calculated by the corresponding probability of each feature separately and multiplying them together. In the multivariate Gaussian distribution model, the covariance matrix of the feature and calculated probability is constructed by substituting all the features into Equation (9).

(9) $$p\left(x\right)=\prod_{j=1}^{n}p\left(x_{j};\mu_{j},\sigma_{j}^{2}\right)=\prod_{j=1}^{n}\frac{1}{\sqrt{2\pi }\sigma_{j}}\exp\left(-\left(\frac{{\left(x_{j}-\mu_{j}\right)}^{2}}{2\sigma^{2}}\right)\right)$$

The ensemble regression model is mainly based on the weighted average. The weighted average is used to represent the final prediction results of all models. Finally, the prediction results need to be rounded to be used as the final prediction classification. When the appropriate weight assignment is found, the negative influence of each other can be eliminated between different algorithms, and a more robust strong learner can be obtained.

**Figure 6:**
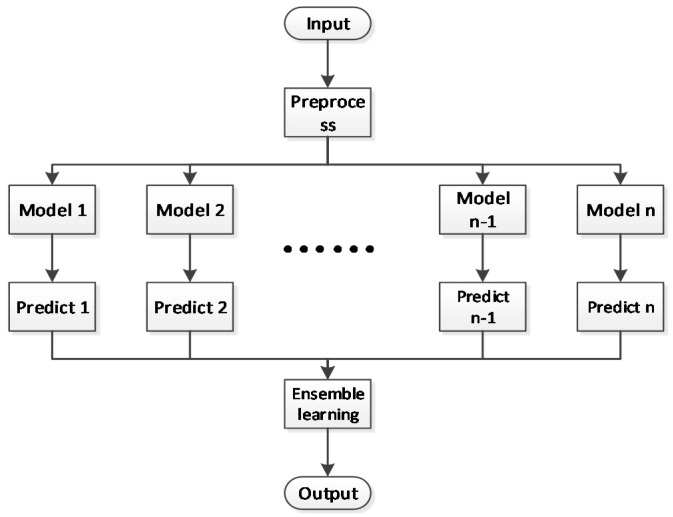
Flowchart of the ensemble learning method.

The ensemble classification model mainly uses the voting method, which is divided into two kinds. One is peer-to-peer voting without weights, that is, each basic model has equal status, and the final result is won by a large number of votes. The other is weighted voting, which assigns different weights to different algorithms. In our paper, we used peer-to-peer voting with the weights method to analyze the relationship among different basic models. The classes are equivalent to the candidates, while the classifiers are equivalent to voters. Each classifier outputs a class equivalent to a vote for the corresponding class, so the class with the most final votes is the final class. In a similar weighted averaging way, the voting scores of each algorithm are equal to their own weights. Finally, the result with large voting scores wins. The flow chart of the ensemble learning method is shown in Figure 6.

## 4. Case Study

In order to improve the data reliability and model practicability of machine olfaction, a novel electronic nose with active perception was manufactured. In the environment of 26 ± 2 °C and 50% ± 5% RH relative humidity, each object was put beside the electronic nose for 3 s and actively adsorbed it via the built-in turbine of the electronic nose. After circuit and program processing, the 300 consecutive sampling points were obtained as a data record. The values of each point are the corresponding voltage signals in units of mV, which reflect the odor data of the object. There were ten classes in the data set, ‘white vinegar’, ‘orange’, ‘water alcohol’, ‘Erguotou’, ‘soy sauce’, ‘kumquat’, ‘tangerine’, ‘cola’, ‘apple’, and ‘Sprite’. Considering that the vibration, electromagnetic interference, and other factors might have influence on the data, the data should be preprocessed first.

### 4.1. Data Processing

After using the active adsorption electronic nose to obtain olfactory data, the data were standardized. Scaling the olfactory data in proportion without changing its distribution characteristics made them fall into the range of size (0, 1) for improving the convergence speed of the model and the accuracy of the model.

Besides, the optimal solutions related to the dimension of data such as SVM do not have an equivalent solution before and after standardization. If not standardized, the parameters of the possible model will be dominated by some larger or smaller data.

Then, fast Fourier transform (FFT) was applied to transform the olfactory data from the time domain to the frequency domain and provide data for subsequent ensemble learning model training.

Meanwhile, the generation of odor is due to the thermal movement of the molecules that make up the object, which diffuses. The diffused molecules are absorbed by the human body and, thus, produce odor [19]. The method of the derivation of the odor change curve was used to eliminate the influence of the concentration on odor types and make the data have better attributes. According to the definition formula of derivative:

(10) $$f^{\prime }\left(x\right)=\underset{\Delta x\to 0}{\lim}\frac{f\left(x+\Delta x\right)-f\left(x\right)}{\Delta x}=\underset{\Delta x\to 0}{\lim}\frac{f\left(x\right)-f\left(x-\Delta x\right)}{\Delta x}\;$$

The derivative of the function is equal to the ratio of the increment in the function (Δ*f*) to the increment in the independent variable (Δ*x*) with Δ*x* approaching zero. We make the interval between two sampling points Δ*x*, and the change in the corresponding value Δ*f* to calculate the derivation.

**Figure 7:**
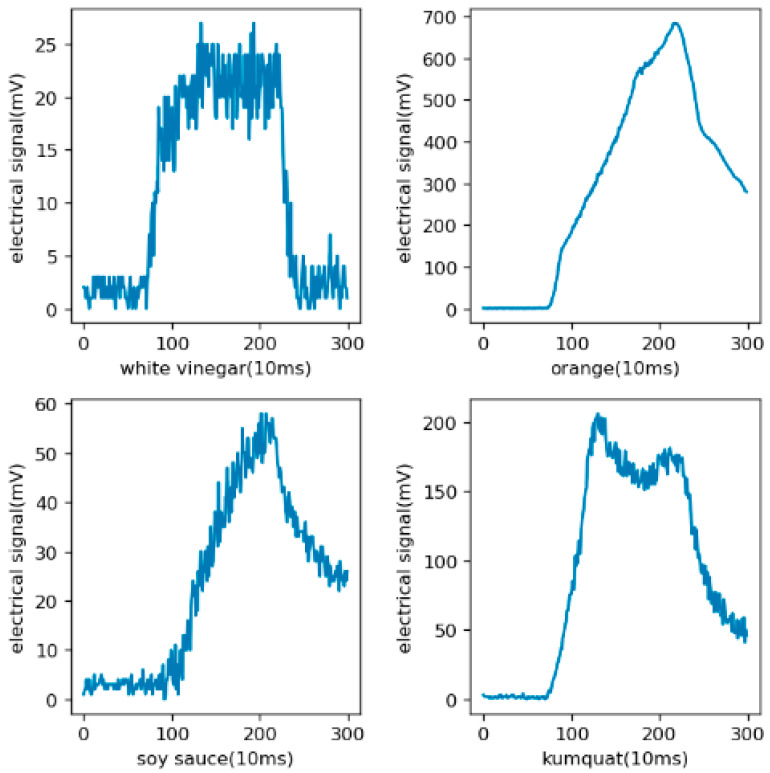
A broken-line diagram of the original data, in which the abscissa is time and the ordinate is the electrical signal with the unit of mV indicating the concentration. In the environment of 26 ± 2 °C and 50% ± 5%RH relative humidity, the E-Nose collects 100 values per second and 300 values at a time.

The distribution of the original data is shown in Figure 7. The electrical signal is from the mean values of the four gas sensors. It can be seen from the figure that there were obvious differences in the odors produced by different kinds of objects. For pungent odors such as alcohol, the proportion of high-concentration areas was higher, which is opposite to the objects with mild odors.

**Figure 8:**
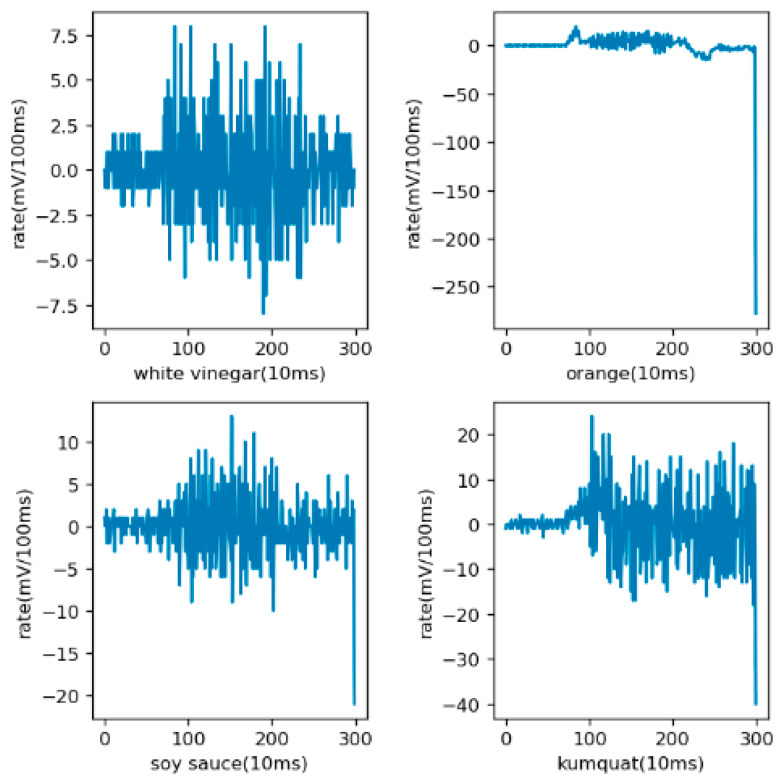
Sample diagram after derivation. The *X*-axis represents sampling time with the unit of 10 ms. The *Y*-axis represents the rate of change of electrical signals.

At the beginning of the training, there was no derivative operation, and the training results were not particularly prominent. After discussion and reflection, the derivative operation was carried out. The reason has been mentioned above. The results of the derivation are shown in Figure 8. From the figure, it can be seen that the difference between the data maps of different objects was more obvious after derivation, and from the training results, derivation was beneficial to the final recognition.

**Figure 9:**
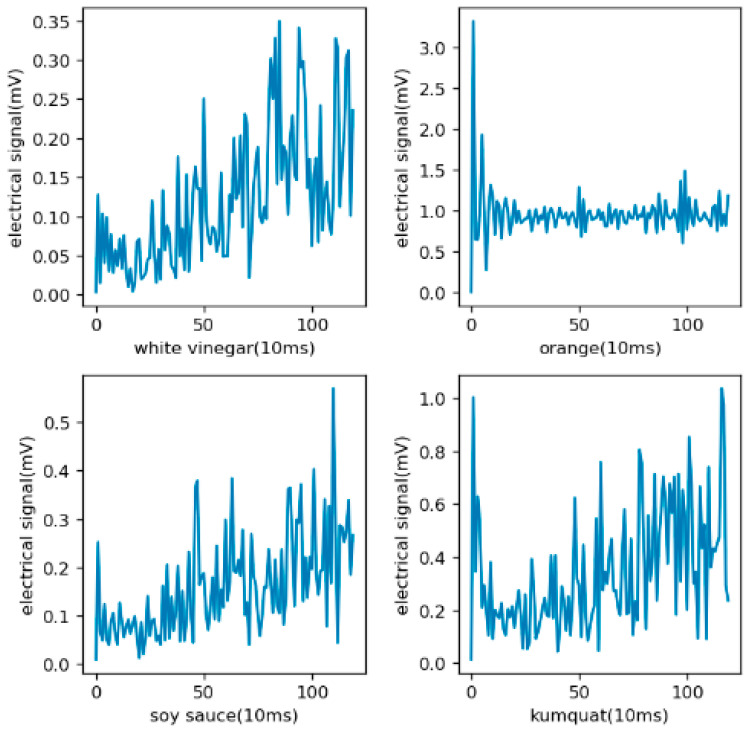
Sample diagram after FFT and normalization. The *X*-axis represents the frequency after FFT with the unit of Hz. The *Y*-axis represents the electrical signal with the unit of mV indicating the concentration.

Then, in order to train better and faster, FFT and normalization were used for the data. The data after operation are shown in Figure 9. After processing, the range of data fluctuation was smaller and the interval length was within 0.8. The processed data provided the supplement for the accuracy of classification methods.

### 4.2. Single Algorithms

First, the processed data were randomly divided into the training set and test set according to the ratio of 8:2 to be prepared for training. This was divided in two ways; one was the regression method, which regards the category as a value and rounds the regression results as the final classification results. The other was to use the classification method directly to obtain the final classification results. The classification accuracy of the test set was taken as the criterion. In order to prove the effect of derivation on the data, the data of derivation and nonderivation were trained.

The regression method used Random Forest Regressor, Gradient Boosting Regressor, Extra Trees Regressor, LGBM Regressor, XGB Regressor, in addition to classified SVC, which are hereafter referred to as RFR, GBR, ETR, LGR, XGR, and SVC, respectively. First, we dealt with the parameters of a single algorithm, and we used GridSearchCV to adjust the parameters so that the single algorithm achieved the best results. Regarding the parameters of a single algorithm, we first initialized the parameters based on experience, and we then fine-tuned them to find their general trend of change. Then, we set a general range and step size based on the trend of change, and then used GridSearchCV to automatically modify the parameters and train the model. Detailed adjustment parameters and alternative values are shown in the Table 1.

**Figure 10:**
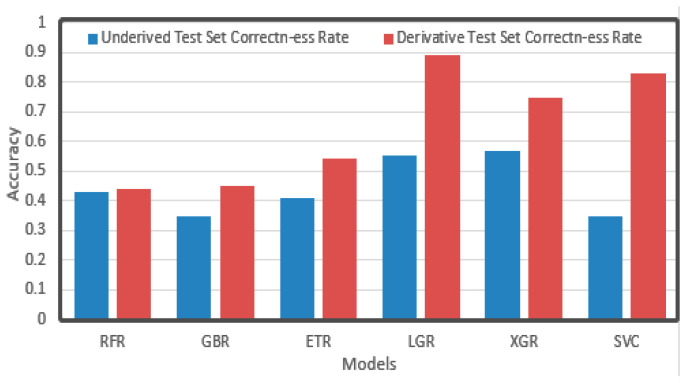
Accuracy of single regression algorithm.

The prediction results are shown in Figure 10. It can be seen that after derivation, the accuracy significantly improved compared with the result before derivation, among which LGR, XGR, and SVC reached relatively good levels. Before and after derivation, the accuracy of RFR did not change significantly, but LGR increased from 0.5 to 0.9, and SVR increased from 0.3 to 0.8, nearly tripling. It may be that derivation reduces the dimensionality of data, which has a greater impact on the dimensionality-related algorithms such as SVR.

Random Forest Classifier, Gradient Boosting Classifier, Decision Tree Classifier, KNeighbors Classifier, LGBM Classifier, XGBC Classifier, SVC, Gauss NB, and Gaussian Process Classifier were used in the classification method. They are hereafter referred to as RFC, GBC, DTC, KNN, LGC, XGC, SVC, GNB, and GPC, respectively. Detailed adjustment parameters and alternative values are shown in the Table 2.

**Figure 11:**
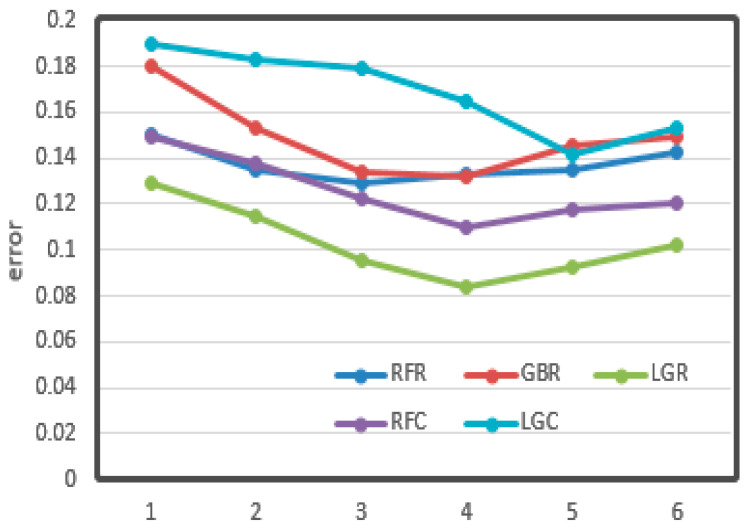
Max depth vs. cv error. The *X*-axis represents the value of max depth. The *Y*-axis represents the cv error.

**Figure 12:**
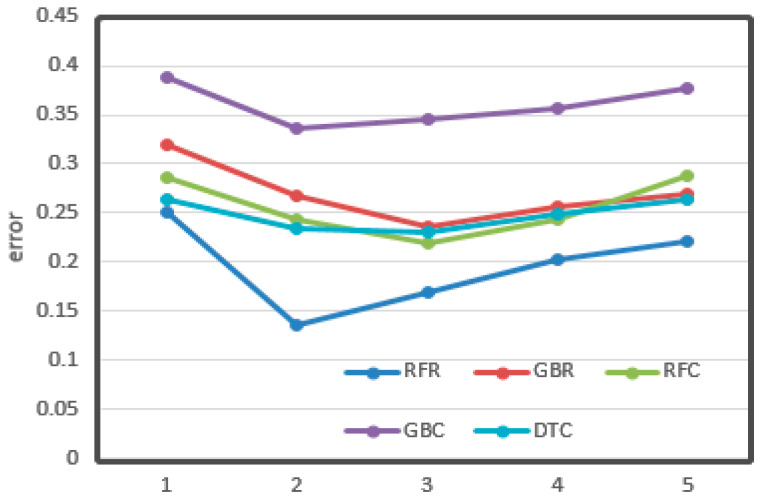
Min sample leaf vs. cv error. The *X*-axis represents the value of min samples. The *Y*-axis represents the cv error.

**Figure 13:**
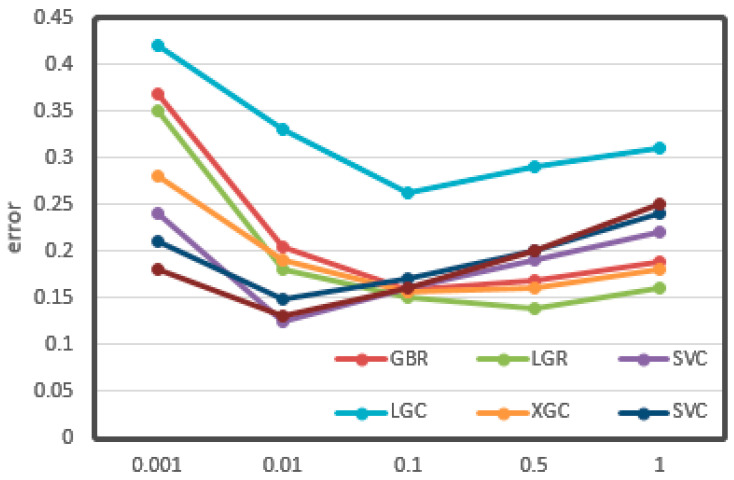
Learning rate vs. cv error. The *X*-axis represents the value of learning rate. The *Y*-axis represents the cv error.

The relationships between the value of max depth, min samples leaf, and learning rate and CV loss are shown in Figure 11, Figure 12 and Figure 13, respectively.

From the figures, it can be seen that some important parameters had considerable influence on the error of the results. In the process of adjusting the max depth, in Figure 11, the error of LGR reduced from 0.12 to 0.08, which decreased by 30%. It also decreased by about 20% overall. The division of the min sample leaf had a great impact on the results. In Figure 12, the RFR with the greatest change decreased from 0.25 to less than 0.15, nearly half of the original. From Figure 13, it can be seen that the learning rate was very important for training. The biggest change of LGR was from 0.35 to below 0.15, which reduced by more than half. These results may be related to the distribution characteristics and data processing methods of odor data. All of the optimal values have been written in the tables above.

**Figure 14:**
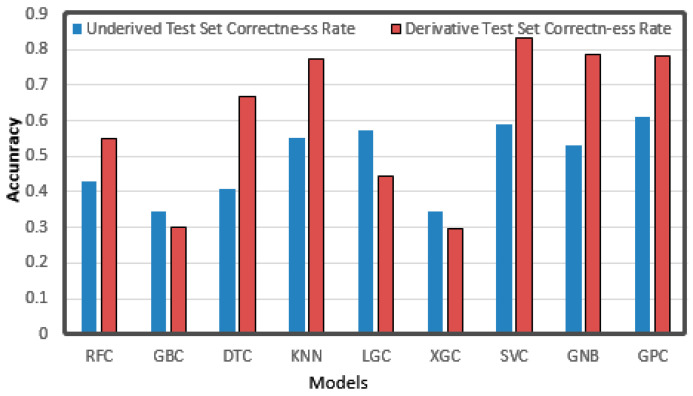
Accuracy of a single classification algorithm.

The forecast results are shown in Figure 14. Most of the algorithms had better results after derivation than before, among which KNN, DTC, SVC, GNB, and GPC achieved good results. We can see that the correct rate of most algorithms improved, among which the DTC with the highest improvement rose from 0.4 to 0.66, and most other algorithms also improved by about 0.2, which shows that the derivation operation was really very helpful. After derivation, the highest correct rate of a single algorithm reached 0.83. However, there are also a few algorithms that had lower accuracies after derivation, and the three reduced algorithms GBC, LGC, and XGC are all boosting-based algorithms. It may be that in the boosting algorithm, Taylor expansion is used to calculate the loss function, and a higher derivative is needed. The Taylor expansion after derivation is one less exponential minimum term than that before derivation. When calculating the error, the exponential minimum term is the maximum error and results in a lower accuracy. Thus, we used the original data to train the boosting class algorithm and used derivation for others.

### 4.3. Ensemble Learning of Regression and Classification

The idea of permutation and combination is used to enumerate all kinds of algorithms and weight combination, and to constantly compare and find the best combination.

**Figure 15:**
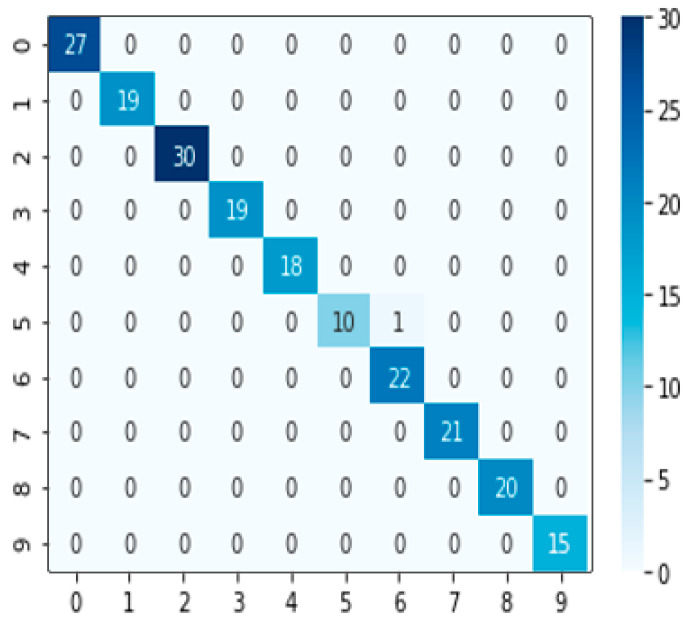
The confusion matrix of SVM, XGB, and LGB integration, with abscissa as the real value and ordinate as the prediction.

In the regression method, three better performance algorithms were selected: SVM, XGB, and LGB. According to the integration method mentioned above, we assigned weights to three models, w_1, w_2, and w_3 respectively. Among them, w_1 + w_2 + w_3 = 1, and the optimal collocation 0.3, 0.45, and 0.25 was found from dozens of weight collocations through permutation and combination to obtain the SXL model. The average training time of each weight collocation was about 10 s. The accuracy of regression under this combination was 0.995049504950495. The confusion matrix is shown in Figure 15. From the figure, it can be seen that in nearly 300 test sets, there was only one result error. The target was classified, which belonged to the fifth category of ‘soy sauce’ into the sixth category of ‘kumquat’. After consulting the data, we found that the pH value of ‘soy sauce’ was 4, and the pH value of ‘kumquat’ was 3. The acidity of ‘soy sauce’ was comparatively similar, possibly due to the reason of H ion concentration. As a result, the data collected by the electrochemical sensor were comparatively similar, leading to classification errors.

In the classification method, KNN, DTC, SVC, GNB, and GPC were chosen. As the single algorithm results of the classification method were worse than those of regression, we adopted more combinations and collocations, and we searched for the optimal weight allocation by the permutation and combination for each combination. The traditional ensemble learning methods include bagging and boosting. As there were three groups indicating the same weight ratio (1:1, 2:2, and 3:3), the number of weight combinations should follow as $3^{n}-2$, where n is the number of the cases.

(1) Two algorithms.

First, the two algorithms were combined. They were SVC + KNN, SVC + GPC, SVC + GNB, SVC + DTC, GNB + DTC, GNB + KNN, GNB + GPC, GPC + KNN, GPC + DTC, and KNN + DTC. Each combination was matched with seven different weight combinations: 1:1, 1:2, 1:3, 2:1, 2:3, 3:1, and 3:2. The average training time of each weight combination was within 10 s. The average training time that contains the gauss process was about 20 s. The final optimal weight allocation and results are shown in Table 3.

From the table, it can be seen that the single classification algorithm could achieve a 0.83 accuracy (SVC). By voting through two or two integrations, the highest correct rate was 0.89 (SVC and GNB, GNB and GPC), which achieved a relatively large improvement. However, the algorithm that includes GPC would take much time to train because of the calculation of partial variance, which is modified in the following part.

(2) Three algorithms.

The combination of three algorithms was used to classify the votes, using the following nine combinations: KNN + GNB + SVC, KNN + GPC + SVC, KNN + DTC + SVC, DTC + GNB + GPC, GPC + GNB + SVC, SVM + GNB + DTC, SVC + GPC + DTC, GNB + GPC + KNN, and GPC + KNN + DTC. Each combination was matched with 25 different weight combinations. That means that there were 9 × 25 = 225 different combinations. The average training time of each weight combination was within 10 s. The average training time that contains the gauss process was about 20 s. The optimal weight distribution and correct rate are shown in Table 4.

The results show that the accuracies of SVC and GNB were highest, reaching 0.91. Compared with the optimal combination of the two algorithms, the accuracy of SVC and GNB improved by 0.02.

(3) Four algorithms.

In the voting classification learning of the four algorithms, the following five combinations were adopted: SVC + GNB + GPC + KNN, SVC + GNB + GPC + DTC, GNB + GPC + KNN + DTC, SVC + GNB + KNN + DTC, SVC + KNN + DTC, SVC + GPC + KNN + DTC, and SVC + GPC + KNN + DTC. Each combination was matched with 79 different weight combinations. The number of different combinations was 7 × 78 = 546 in total. The average training time of each weight combination was within 10 s. The average training time that contains the gauss process was about 20 s. The optimal weight distribution and accuracy are shown in Table 5.

Among the five combinations, the optimal result was 0.96 (SVC, GNB, GPC, and DTC). Compared with the previous combination, it had a very high improvement. However, with the increase in the number of algorithms, and with the addition of Gaussian process, the operation process was gradually slowed down and the efficiency was gradually reduced, so no more algorithm integration was needed.

### 4.4. Small Size Data Prediction

**Figure 16:**
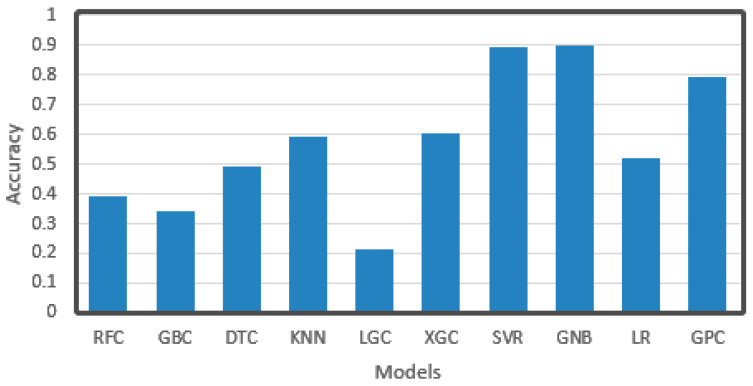
Accuracy of training a single classification model with only 30% data.

In order to solve the problem of slow calculation mentioned in the previous part, we selected a small part of the training set for training (30%) to test the performance of each algorithm. As we have come to the conclusion that derivation will lead to better results, the original data without derivation were no longer trained. These data were pre-processed, and then each classifier was trained and used to predict the data of the test set. The results are shown in Figure 16. As can be seen from the figure, the accuracy of RFC, DTC, and other algorithms based on decision tree were all below 0.5, and the clustering KNN was only up to 0.6, while GNB and GPC based on the gauss process were both above 0.8, and GNB even achieved the highest level of 0.9. At the same time, SVR ranked second place around 0.9. This may be because algorithms such as decision tree clustering need multiple trainings of a large amount of data to obtain a better model, while the gauss process is based on the statistical model, which has a higher utilization rate of data and can better match the original distribution of data, and has a better effect for small data. SVR is sensitive to the distribution of data, which may be related to the distribution of original data and the way of data preprocessing.

After that, we classified the votes and voted on the three better algorithms. In this voting, we used the following combinations: GPC + SVR + GNB, GPC + SVR, GPC + GNB, and SVR + GNB. The first combination was matched with 25 different weight combinations. The rest was matched with seven different weight combinations. The number of different combinations was 25 + 7 × 3 = 46 in total. The average training time of each combination of weight was about 20 s. The algorithm model based on the Gaussian process requires complex calculation of the data based on covariance, so the training speed might be relatively slow, but the time of data acquisition could be reduced by nearly 20 times, and the overall speed improved. The results are shown in Table 6.

As it can be seen, the algorithm with the highest accuracy, GSG, achieved an accuracy rate of 0.996, which is quite remarkable when only 30% data were used. Some combinations only reached 0.6, which is not as good as the results of a single algorithm, indicating that the wrong combinations between different algorithms may achieve the opposite effect. After a lot of experiments, we finally obtained the GSG algorithm.

Combining active electronic nose and the AGSG algorithm, the training data requirement was reduced by 70%, the single data acquisition time was reduced ten times, the target recognition accuracy rate was 0.996, and the basic functions of mobile robot were realized.

## 5. Conclusions

Machine olfaction plays a key role in the development of robots. Target recognition through olfaction will also be a key point in the future development of robots. In this paper, a novel electronic nose with active perception based on an ensemble learning method was designed and verified. The designed electronic nose cannot only realize active perception with different smells, but also reduce the time of data acquisition. The active adsorption electronic nose is based on the turbine system, which reduces the odor collection time by an order of ten. Even for the small size of data (30% training data) combining the active electronic nose and the AGSG algorithm, the single data acquisition time was reduced by a factor of ten, the target recognition accuracy rate was 0.996, and the basic functions of the mobile robot were realized. The active perception electronic nose and the AGSG algorithm were applied to the robot so that we could realize a complete mobile robot with machine olfactory function.

In the future, the dataset should be expanded to allow robots to recognize more targets. In addition, the robot’s electronic nose should be flexible so that when recognizing farther or higher objects, we can extend the ‘nose’ to ‘sniff’. Besides, we are also going to combine traditional target recognition, add ‘eyes’ to the robot, and use a combination of olfaction and vision to identify objects and even external environments. This kind of robot can be used to explore unknown areas in various harsh environments, such as underground or for deep-sea analysis of odor components.

## Figures and Tables

**Table 1:** Parameter adjustment of regression models with GridSearchCV.

Model	Parameters	Alternative Values	Optimal Value
RFR	Estimators	120, 300, 500, 800	300
	Max depth	1, 2, 3, 4, 5, 6	3
	Min samples split	1, 2, 3, 4, 5	2
	Min samples leaf	1, 2, 5, 10	5
GBR	Learning rate	0.001, 0.01, 0.5, 0.1, 1	0.1
	Estimators	50, 100, 150, 200	100
	Min samples split	2, 4, 6	4
	Min samples leaf	1, 2, 3, 4, 5	3
	Max depth	1, 2, 3, 4, 5, 6	4
LGR	Leaves num	20, 25, 30, 35	30
	Learning_rate	0.001, 0.01, 0.5, 0.1, 1	0.5
	Max depth	1, 2, 3, 4, 5, 6	4
SVC	C	0.001, 0.01, 0.5, 0.1, 1	0.1
	Gamma	‘auto’, ‘RS’	‘auto’
	Class weight	‘Balanced’, None	None

‘C’ means penalty coefficient of error Item.

**Table 2:** Parameter adjustment of classification models with GridSearchCV.

Model	Parameters	Alternative Values	Optimal Value
RFC	Estimators	120, 300, 500, 800	300
	Max depth	1, 2, 3, 4, 5, 6	4
	Min samples split	1, 2, 5, 10, 15, 100	5
	Min samples leaf	1, 2, 3, 4, 5	3
GBC	Learning rate	0.001, 0.01, 0.5, 0.1, 1	0.1
	Estimators	50, 100, 150, 200	100
	Min samples split	2, 4, 6	2
	Min samples leaf	1, 2, 3, 4, 5	2
	Max depth	3, 4, 5, 6	6
DTC	Min samples split	1, 2, 3, 4, 5	1
	Min samples leaf	1, 2, 3, 4, 5	3
LGC	Leaves num	20, 25, 30, 35	30
	Learning_rate	0.001, 0.01, 0.5, 0.1, 1	0.1
	Max depth	1, 2, 3, 4, 5, 6	5
XGC	Min child weight	1, 2, 3, 4, 5	2
	Subsample	1, 2, 3	1
	Learning rate	0.001, 0.01, 0.5, 0.1, 1	0.01
SVC	C	0.001, 0.01, 0.5, 0.1, 1	0.01
	Gamma	‘auto’, ‘RS’	‘auto’
	Class weight	‘Balanced’, None	None
GPC	Alpha	1 × 10^−10^, 1 × 10^−9^, 1 × 10^−8^, 1 × 10^−7^	1.00 × 10^−9^

**Table 3:** Ensemble learning of two classification algorithms.

Model 1	Model 2	$w_{1}$	$w_{2}$	Accuracy
SVC	KNN	1	1	0.772
SVC	GPC	3	1	0.806
SVC	GNB	3	2	0.891
SVC	DTC	3	1	0.806
GNB	DTC	1	1	0.732
GNB	KNN	1	1	0.787
GNB	GPC	3	1	0.891
GPC	KNN	1	1	0.772
GPC	DTC	3	1	0.668
KNN	DTC	2	2	0.816

**Table 4:** Ensemble learning of three classification algorithms.

Model 1	Model 2	Model 3	$w_{1}$	$w_{2}$	$w_{3}$	Accuracy
KNN	GNB	SVC	1	3	3	0.891
KNN	GPC	SVC	1	1	1	0.891
KNN	DTC	SVC	2	1	2	0.851
DTC	GNB	GPC	2	1	3	0.891
GPC	GNB	SVC	2	1	3	0.846
SVC	GNB	DTC	3	3	3	0.910
SVC	GPC	DTC	2	2	2	0.866
GNB	GPC	KNN	2	3	1	0.891
GPC	KNN	DTC	2	2	2	0.792

**Table 5:** Ensemble learning of four classification algorithms.

Model 1	Model 2	Model 3	Model 4	$w_{1}$	$w_{2}$	$w_{3}$	$w_{4}$	Accuracy
SVC	GNB	GPC	KNN	1	2	1	1	0.891
SVC	GNB	GPC	DTC	3	3	1	3	0.960
GNB	GPC	KNN	DTC	3	1	2	1	0.891
SVC	GNB	KNN	DTC	3	1	3	1	0.891
SVC	GPC	KNN	DTC	2	2	1	3	0.856

**Table 6:** Accuracy of the model by integrating 2–3 algorithms based on Gaussian process.

Model 1	Model 2	Model 3	$w_{1}$	$w_{2}$	$w_{3}$	Accuracy
GPC	SVR	GNB	3	1	1	0.996
GPC	SVR		2	1		0.910
GPC	GNB		1	1		0.603
SVR	GNB		3	1		0.603

## Data Availability

The datasets used or analyzed during the current study are available from the authors on reasonable request.
